# The Pathology and Treatment of Hemorrhoids

**Published:** 1854-08

**Authors:** T. G. Richardson

**Affiliations:** Demonstrator of Anatomy in the University of Louisville; Louisville


### ART. IV.—THE PATHOLOGY AND TREATMENT OF HEMORRHOIDS.

BY T. G. RICHARDSON, M. D., DEMONSTRATOR OF ANATOMY IN THE UNIVERSITY OF LOUISVILLE.

ONE of the most common, and by no means the least troublesome, of the numerous diseases to which the rectum is liable, is that, the title of which stands at the head of this article; and considering its exceeding frequency and its great importance in a practical point of view, it is somewhat remarkable how lightly it is treated of in most of the text-books on general surgery, and how loose and unsatisfactory are the ideas of a majority of even the best practitioners of the country in regard both to its causes and nature, and the treatment required for its relief. It is with the hope of being able to put some of the readers of the Journal in possession of facts which the too slender libraries of many of them do not furnish, that I take the liberty of presenting them with the results of my observations and study.

*General Considerations.*—Under the common term *Hemorrhoids* or *Piles*, are included two entirely distinct and separate affections, distinguished ordinarily by the prefixes 'external,' and 'internal,' or 'blind,' and 'bleeding' piles. It is true that they are both bloody tumors, produced by a modification of the same causes, often co-exist, and are situated in adjacent parts of the same organ; but, as will be presently pointed out, they are totally different in their anatomy, in their course and consequences, and in the treatment demanded for their cure. The fact that they are so entirely dissimilar should be distinctly borne in mind, in order to avoid much of that practical confusion that seems to prevail, not only among physicians generally, but even among many distinguished lecturers and writers on surgery. That such confusion does exist one has only to take up and examine all the more familiar American and foreign books in which the subject is treated of, to be fully satisfied. In prosecuting some inquiries lately in regard to diseases of the rectum, I have had occasion to look over quite a number of publications of this sort, besides others of a more special character, and I must confess that, with one exception, I have found no clear and satisfactory recognition of the great distinction that it is so absolutely necessary to make between these diseases. The exception is GROSS's Pathological Anatomy, wherein the celebrated author draws particular attention to the point in question, and leaves little or nothing to be desired in the way of an accurate and well-defined description of, and discrimination between, the morbid anatomy of the two. The great majority of other writers acknowledge that there is a difference, but do not tell us precisely what it is, or at least fail to impress it upon the reader in such a manner as to make him feel its importance either in a pathological or a practical point of view. Others again (but fortunately very few), and among them the world-renowned ABERNETHY, deny that there is any difference in structure, and consequently make none in practice.

In the following imperfect outline of the two affections, I have attempted to arrange their several characters in a sort of parallel light, so that their distinguishing marks may be more prominently brought into view than could have been done by giving a separate and continuous description of each.

1. *Diagnosis.—External Piles* are exceedingly common, occur for the most part about the middle period of life, although occasionally observed in young children,* are of exceeding frequency in females during parturition and the latter months of pregnancy, and prevail probably to a greater extent in Southern than in Northern latitudes. They are situated at the *verge of the anus*, just where the skin of the nates and mucous membrane of the rectum become blended together, where they form tolerably firm, elastic, prominent, well-defined tumors of a brownish-red or purplish hue. They are generally multiple, often numbering as many as five or six, although frequently single, especially when large, and vary in size, from that of a medium shot to that of a large filbert. They are usually produced suddenly during parturition or violent straining at stool, and are generally attended, for a few days, with more or less pain, often of an acute character, and an annoying feeling as though a large foreign body were lodged between the nates. They are rarely if ever the seat of hemorrhage amounting to more than a few drachms, and only so when accidentally opened by rupture or ulceration.

*Internal, or bleeding Piles* are far less frequent than the external variety, seldom if ever occur as an uncomplicated affection before the age of twenty or twenty-five, and are, in this country, according to my own observations and inquiries, much more common among men than women. This last statement is, I am aware, at variance with the published authorities generally upon the subject, but not, as will be hereafter shown, with the nature of the causes producing the complaint. The only author to whom I can refer at this moment as having observed the same fact, is M. VIDAL, who states in his "*Pathologie Externe*" that it is remarkable how few females, excepting those who have frequently borne children, are found to labor under tumors of this sort.

*Internal Hemorrhoids* are situated a short distance within the rectum, immediately above the internal sphincter muscle, and may be felt by the introduction of the finger as smooth, spongy, undefined swellings occupying the space of an inch or two in an upward direction, and often the whole circumference of the bowel. When

* Gross's Pathological Anatomy.

made to "come down" below the sphincter, as they nearly always are by violent efforts at evacuation, and as they always should be, if possible, when an examination is desired, they become erect and turgid with blood, and being partially strangulated by the contraction of the muscle just mentioned, present themselves externally in the form of large rounded tumors, of an intensely brownish-red or livid color, continuous with each other at their bases, and often so crowded together as to have the appearance of one large irregular mass, not unlike, and often mistaken for, prolapsus of the rectum. They are of comparatively slow growth, and generally multiple, the number varying from two or three to six or eight, although, as just intimated, it is frequently almost impossible to discover their separating lines. When thus protruded they are usually the seat of a good deal of bearing-down pain, sometimes sufficient to produce fainting; and under these circumstanstances they are also exceedingly liable to hemorrhage from rough handling or simply from over-distension of the vessels. The amount of bleeding varies from a few drops to several ounces, and being habitual in many persons, frequently brings on, and keeps up a state of general anemia, by which the constitution is gradually, but surely undermined, and the life of the individual placed in imminent jeopardy.

In suspected cases where there is no protrusion, the existence of the disease is best ascertained by the use of a rectal speculum, consisting of a solid glass or metallic cylinder, with an oblong opening upon one side near the distal extremity.

*Pathological Anatomy.—External Piles* are formed by the rupture of one or more of the small veins that ramify about the verge of the anus. They consist, therefore, simply of a clot of blood occupying a corresponding cavity which it forms for itself in the loose areolar tissue of the part, and are covered on the lower side by the skin, and on the upper by the mucous membrane of the rectum. That this is the whole anatomy of tumors of this variety soon after their formation, I have fully satisfied myself by frequent dissection, thus verifying the observations of Prof. GROSS upon the same point.

*Internal Piles* belong to the class of erectile tumors, and consist essentially of masses of irregularly dilated, tortuous, and convoluted capillaries, mostly venous, occupying the submucous areolar tissue of the lower part of the rectum. They are in fact nearly similar, in every respect, except in the mode of their production, to *nevus* or aneurism by anastomosis, and are hence sometimes called varicose hemorrhoids. Like nevi, they are, when in a state of rest, soft, flaccid, and spongy to the touch, but in consequence of pressure on their venous trunks in violent straining, become swollen and tense, are generally forced out beyond the sphincter, and as before remarked, if cut or scratched, or even roughly handled while in this state of turgescence or erection, bleed, sometimes so profusely as to produce syncope, and occasionally endanger the life of the individual. In most cases the mucous membrane covering the congeries of vessels is thin and delicate, but in consesequence of frequent protrusion and handling it may take on a condition of chronic inflammation producing thickening and induration of its structure, and the discharge of a large quantity of stringy mucus mixed with more or less pus and blood.

2. *Course and Consequences.—External Hemorrhoids* give rise, when first formed, to more or less inflammation attended with a good deal of local distress, and occasionally a little sympathetic fever. In a few rare instances the inflammation proceeds to suppuration and the formation of abscess, which, upon being opened, is followed by a discharge of the coagulated blood along with the matter, and thus produces a spontaneous cure. Ordinarily, however, the inflammatory action subsides in a day or two, and the tumors, if let alone, undergo in the course of time a very gradual but material change in their structure. This change consists in the absorption of the watery and coloring matters of the effused blood, and the organization of its fibrin, by which means the tumors are very much diminished in size, lose their reddish or livid color, and their acute sensibility, and remain as permanent appendages to the margin of the anus, in the form of little pendulous excrescences of a pale appearance and flabby consistence. It is not uncommon to find a row of these appendages encircling the anal orifice, where they may give rise to no inconsiderable annoyance by friction against each other and between the nates in walking, especially in hot weather. Sometimes in consequence of this chafing, and the want of cleanliness, they become the seat of a most painful ulceration upon their inner or mucous surfaces, and are said also to occasionally degenerate into malignant disease; but of this latter I have never witnessed an instance.

*Internal Hemorrhoids* occasionally, but very rarely, undergo a spontaneous cure. This may take place in one or two ways: 1st. From negligence or some other cause the tumors are not returned as soon as they should be, after defecation; inflammation is set up, the sphincter muscle becomes spasmodically contracted, and the protruded parts fall into a state of mortification in consequence of the strangulation. In this manner the whole of the diseased mass may slough off, and if the patient's constitution is able to bear up against this new malady he will be permanently cured of the old. Two cases of this sort are reported by Mr. HOWSHIP, and one came to my own knowledge in an adjacent state not many weeks ago. In the second place, inflammation may, from some cause or other, be lighted up in the diseased part, attended with coagulation of the blood, and effusion of lymph, by which means the vessels are obliterated, and in the course of time the coagulated blood and effused lymph becoming absorbed, the tumors will be found to have entirely disappeared.

Most frequently, however, the disease after having become quite bad, either remains stationary for sometime, or progresses steadily from bad to worse, the tumors often attaining almost the size of a man's fist. Under these circumstances the suffering that they give rise to is very great. One individual with whom I am acquainted, and who has pertinaciously declined all surgical interference, is obliged to provide himself regularly every morning when he goes to stool, with towels, soft cloths, &c., for the purpose of cleanliness, and having emptied his bowels and washed the parts in cold water, has to get upon his knees and one hand, while with the other he gradually effects reduction; and such is the sense of fatigue and exhaustion after the operation is over, that he has invariably to lie down for an hour or two before he recovers from its effects.

But the most serious consequence attending the disease, is the general anemia resulting from the frequent losses of blood. I cannot better describe this condition than by making use of the language of Mr. SYME.* "The patient loses flesh and acquires

* Diseases of the Rectum.

a remarkable paleness of complexion which is afterwards exchanged for a peculiar dingy-yellow hue like that of imperfectly bleached wax. The lips no longer possess their vermillion color, but resemble those of a dead body; the tongue, too, has a blanched appearance very characteristic of the state induced by excessive or continued depletion. These symptoms are attended with great listlessness or want of energy, both of body and mind, disturbed sleep, irritability of temper, quick pulse and head-ache, which is generally increased by rising up more than by lying down. Palpitation and pain in the region of the heart, and difficulty of breathing, are also frequently induced by slight exertion or agitation of any kind." And what is remarkable, these symptoms sometimes exist without the patient's knowledge of their true cause. A case of this kind came under my observation a few years ago in which the individual ascribed his symptoms to disorder of his stomach. Although he had often noticed that his fœces were streaked with blood, and that blood appeared upon the paper with which he wiped himself, he was not aware that he was laboring under hemorrhoids, the tumors having never, to his knowledge, protruded at the anus. Indeed, I may remark, that he had been treated for dyspepsia, and would in all probability have died under this impression had he not fallen into other hands, and been relieved of the real source of his difficulty. Mr. SYME also mentions a case of the same kind, in which "the patient was supposed to labor under disease of the heart, and whose waxy look, bloodless lips, and defective energy, together with irregular action of the heart, certainly afforded considerable ground for this opinion, but Dr. ALEXANDER discovered that there was an internal hemorrhoid which bled profusely every time the patient went to stool, and I removed it with the effect of quickly restoring him to health.*

3. *Causes.*—The causes of hemorrhoids are predisposing and exciting, of which the former are of more importance in the production of the internal, and the latter of the external variety. The *predisposing causes* are a relaxed phlegmatic temperament, luxurious living, indolent habits, disease of the liver or lungs, and an occupation requiring long standing. Of these I am inclined to think that the last mentioned is the most powerful, and it is, of course,

Op. Cit.

doubly so when combined with one or more of the others. Space will not permit me to enter into an account of the manner in which these causes predispose to the occurrence of piles, but suffice it to say that they are not unfrequently the only assignable causes of the internal variety, but not of the external. The *exciting causes* of *external piles* consist exclusively of such things as interpose a sudden interruption to the return of the venous blood from the hemorrhoidal veins, as, for instance, violent efforts in evacuating the bowels or bladder, parturition, lifting heavy weighs, &c. Their mode of action is very easily understood. The veins of the lower part of the rectum and verge of the anus are very numerous and tortuous, and communicate on the one hand with the portal circle through the superior hemorrhoidal vein, which terminates in the inferior nusenteric, and on the other with the general venous system by a much smaller trunk, called the middle hemorrhoidal, which opens into the internal iliac. Now it is a fact known to almost every one, that in the contraction of the abdominal muscles the diagonal of all the forces is in the direction of the bottom of the pelvis, so that the contents of the abdomen are made to press forcibly upon those of the pelvis, and consequently upon these two venous trunks, but more especially the larger one. If this pressure occur in an individual in whom, from predisposition or habit, the abdominal veins are in a state of unnatural fullness from want of tonicity, excessive alimentation or what not, one of two things occurs; either the hemorrhoidal veins simply dilate, or else one or more of them give way. In the latter case an effusion of blood takes place constituting external hemorrhoids.

The exciting causes of *internal hemorrhoids* may be the same as those concerned in the production of the preceding variety, with some modification in their mode of action. Thus, as just stated, external piles result suddenly from over distension of the veins of the rectum, but for the production of the internal form it is necessary that this interruption should often recur before the venous radicles can be brought into a permanently dilated or varicose state. Where the interruption is habitual and occasionally very great, both forms of the disease are apt to be the result.

But there is another set of causes upon which some authors place no little stress, and which are no doubt very active. They consist of local irritations of any kind whatever, by which an unnatural amount of blood is determined to the rectum. The most common causes of this kind are the presence of foreign bodies and ascarides, riding upon horseback, the habitual use of drastic purgatives, more particularly aloes, other diseases of the rectum, such as chronic dysentery, ulceration, &c., and lastly diseases of adjacent parts, as, for instance, of the uterus and the other genito-urinary organs. It is very easy to understand how such causes might induce the disease in a person already predisposed, and even when there is no very great predisposition, if the irritation have the effect of producing frequent and violent efforts at expulsion. In the latter case, however, the irritation would be properly considered as a predisposing, and the straining the immediate or exciting cause.

4. *Treatment.*—The treatment of hemorrhoids is pallative, radical and preventive; but as it differs very materially in the two varieties, a separate description becomes necessary.

*External Hemorrhoids.*—The *palliative treatment* of external hemorrhoids is called for only when a high degree of inflammatory action attends the disease, or when from fear of the knife, an unwillingness to undergo the necessary exposure, or from some other cause the patient refuses to submit to the radical treatment. It consists simply in the administration of a little cathartic medicine and the local application of cold water for a day or two, followed by some anodyne ointment to allay irritation and prevent friction in walking. A favorite prescription of many practitioners, and one that has been successively transmitted as a sort of professional heir-loom from one physician to another for several generations, is the comp. ointment of nut-galls, composed of equal parts of powdered galls, opium, and lard or simple cerate. The probability is that the great virtue that has been ascribed to this unguent resides more in the lard, which prevents chafing, than in either of the other ingredients. If the inflammation be very considerable, threatening suppuration, the case has to be treated upon the ordinary antiphlogistic plan.

The *radical treatment* is so simple, so satisfactory, and so universally applicable that it should be almost always insisted upon. It consists either in excision or incision. *Excision* is more particularly applicable to cases of long standing, in which the tumors are converted into fleshy appendages. It is performed by taking hold of the part with the fingers or a pair of forceps, and snipping it off at the base, with a pair of blunt-pointed scissors. The operation is attended with intense but momentary pain, but rarely with hemorrhage. *Incision* is the proper operation in all recent cases. It is performed by plunging a sharp-pointed bistoury or an ordinary thumb-lancet into the interior of the tumor and cutting freely from within outward; or it may be just as easily performed with a common scalpel, cutting from without inward. After the incision is made, gentle pressure on each side will cause the clot of blood to roll out, generally in the form of a rounded shot or bullit-like mass. The only subsequent mesures that are necessary are daily ablution in cold water, the application of simple cerate or lard to prevent undue chafing, and the institution of the preventive treatment. I have never witnessed any hemorrhage after the operation beyond a few drachms, and indeed, generally only to the extent of a few drops; if, however, such an accident should occur, it could be easily remedied by the application of pressure or touching the part with a heated probe.

The *preventive treatment* of external piles consists, of course, in the avoidance of all predisposing and exciting causes, more especially the latter, of which a constipated condition of the bowels is one of the most important. To remedy this the patient should be directed to take a sufficient amount of out-door exercise every day; live principally upon gruel, molasses, stale bread, crackers, soup, and such other substances as are known to favor a liquid condition of the fœces; and, if necessary, the frequent and sometimes daily use of mild laxatives, such as sulphur-water, seidlitz powders, citrate of magnesia, extract of black-walnut, &c.

*Internal Hemorrhoids.*—The treatment of internal piles may also be divided into palliative, radical, and preventive.

1. The *palliative treatment* is applicable to a comparatively small number of cases, the majority admitting of radical cure. Where, however, there is serious disease of the liver or lungs, where the patient is very old and has been laboring under the disease a long time without any apparently bad effect upon the constitution, and in cases occurring in the latter months of pregnancy, palliation is all that should be attempted. The indications are to relieve, as much as possible, the pain attendant upon defecation, prevent farther increase of the disease, and restrain hemorrhage, all of which may be fulfilled by the following means: 1st. an abandonment of all predisposing and exciting causes ; 2nd. the use of certain measures to keep the contents of the larger bowels in a liquid or semi-liquid state; 3d. attention to the mode of defecation; 4th. the local application of astringents.

It is hardly necessary to insist upon the first of these means, the removal of all premisposing and excitiog causes. If the patient's occupation be one that requires long standing it should be changed for a more active mode of life; if the constitution be relaxed and debilitated, tonics, a well selected but liberal diet, out-door exercise, &c., will be called for; if the individual has been accustomed to an idle luxurious life, with intemperance in eating and drinking, this must be abandoned, &c., &c.

Attention to the state of the bowels is of very great importance, and the means pointed out in connexion with the preventive treatment of external piles should always be put in practice.

The manner of emptying the bowels requires particular notice. One of the most powerful causes tending to keep up and increase either one of the varieties of hemorrhoidal disease, but more especially the internal, is the habit of stooping down at stool. This is probably oftener the case in the country, where the use of water-closets is so frequently dispensed with by men, but is also true in a less degree even when apartments of this sort are resorted to, owing to an ill-construction of the seat. It is a fact well known, to professional men at least, that the less the body is doubled up in the act of defecation the less the straining or expulsive effort, and consequently the less apt there is of protrusion of hemorrhoidal tumors, if these exist. This is a very important consideration, for the oftener and greater the protrusion the greater the liability to an increase of the disease, and the more exposed the individual is to hemorrhage and pain. The patient should therefore be made to pass his fœces either in the recumbent, or as nearly the erect posture as possible. The latter is more convenient, and less disagreeable to one's sense of decency, and may be accomplished by having a privy constructed with the seat high, say nearly upon a level with the buttocks, and inclining from behind downward and forward, at an angle of about forty-five degrees. Indeed it would be a good precaution for all persons predisposed to this disease to make use of a privy constructed upon this plan. It is a matter too little thought of, but not the less important on that account.

The fourth consideration is the local use of astringents. These are most conveniently employed in the form of washes, in which the parts may be bathed after defecation, or which may be injected into the bowels once or twice a day. For the former purpose the individual should provide himself with a basin of cold water every time he goes to stool, and, when the act of defecation is finished, bathe the parts by sitting over the basin and dashing the water upon them, and then carefully return them. If something more astringent is required, a little alum, or tannin, or sulphate of zinc may be dissolved in the water, or a decoction of oak-bark used in its stead. In cases where the tumors do not protrude, the bowel should be washed out by one or other of these substances after every operation. Occasionally great good is accomplished, and it is said that cases have been cured, by injecting into the rectum at night just before going to bed a small quantity of some powerful astringent, and allowing it to remain in contact with the parts as long as the patient can retain it. For this purpose nothing is better than a strong solution of tannin, or alum, or a dilute solution of muriated tincture of iron.

Medicines given by the mouth are sometimes employed in the treatment of internal piles. Of these the compound confection of pepper, commonly called *Ward's paste*, has obtained a very extensive reputation. SIR. B. BRODIE, than whom there is probably no better authority in surgery now living, thus speaks of it. "There is a medicine that is very often useful in cases where simple expedients fail, namely, the *confectio-piperis composita*, which is similar to what was once very celebrated as Ward's paste. It is like eating coarse ginger-bread; it may be a little disagreeable to be taken, but still it may be taken easily enough; and the patient must persevere in its use for a considerable time. Very severe cases of piles are sometimes cured by it. A lady came to me with one of the worst cases of this disease that I ever saw; the piles were so large, and protruded so constantly, that I did not think there was any chance of curing her, except by the operation hereafter to be described, and I advised her to submit to it. She said the piles made her miserable, and she should be very glad to be cured on any terms, but she was compelled to pay a visit in the country which would render it necessary to delay it for a month. I thought the delay for a month could not hurt her, and under these circumstances I recommended her to give Ward's paste a trial, and see what it would do for her. I heard nothing more of her for six or eight weeks, when she came back, and said she was happy to inform me that she had taken the paste regularly and was now quite well. * * * * How does the Ward's paste operate? It is evident that except any small portion which may be digested it passes into the colon and must become blended with the fœces; and I suspect that thus coming in contact with the piles, it acts upon them as a local application, much as *vinum opii* would act upon the vessels of the conjunctiva in chronic opthalmia." Mr. BRODIE also mentions a case of SIR. E. HOME's, in which the paste was, by mistake, introduced in large quantity into the rectum with the effect of curing the patient.

When internal piles, from any cause whatever, become inflamed and swollen, the treatment for the time being is necessarily directed to subduing the excited action. This accident generally occurs when the tumors are protruded, and the first thing to be done in a case of this kind is to attempt to return them, which is done by first gently pressing the blood out of them and then pushing them up. Subsequently the application of leeches around the anus and the injection of cold water is in general all that is necessary. If however, the efforts to reduce them prove unavailing the only thing that can be done is to puncture them with a sharp-pointed bistoury, and apply cloths wrung out of warm water, and then make another attempt to return them; if still unsuccessful the probability is that mortification will take place, which should be treated in the same manner as mortification in any other part of the body. As heretofore stated, if the patient's constitution is able to sustain this morbid action a cure of the original disease is the result.

2. *Radical Treatment.*—For the radical cure of internal or varicose piles, one of two things is necessary; either the tumors must be removed by an operation, or obliterated by the coagulation of their contents and the effusion of lymph. Removal may be effected by the application of caustics, by excision, or by the ligature; coagulation and adhesive inflammation, by the seton, acupuncturation with hot needles, or injection of irritating fluids.

*Caustics.*—The old practice of destroying hemorrhoidal tumors by the actual cautery, vienna paste, or caustic potash, is pursued by very few intelligent surgeons of the present day, and should indeed be looked upon as an almost obsolete procedure. Indeed, the only distinguished exception that I know of, is the present Baron BOYER, of Paris, who is in the constant habit of using the hot iron in the treatment of this disease. A modification of the caustic plan, however, has been put in practice by Mr. HOUSTON of Dublin, and occasionally also employed by other surgeons with sufficient success to warrant its adoption in cases in which, for some cause or other, the ligature is contra-indicated. It consists in the repeated application of strong nitric acid to one or more of the tumors at a time, for the purpose, not so much of producing a slough, as of exciting a moderate degree of inflammation and consequent coagulation of the contained blood and effusion of lymph. It belongs therefore more properly to the second class of remedies mentioned above. The acid should not be applied to more than two or three of the tumors at once, and not over their whole surface, but only to the extent of about a five-cent piece, and should be immediately followed by a free inunction of the parts with sweet oil or lard, to prevent its contact with the surrounding structures. The application has generally to be repeated from three to five times, with an interval of five or six days between each, and requires no little caution in its management. I have had no experience with it myself, but Prof. GROSS informs me that he has employed it once and with the most satisfactory result.

*Excision.*—At the present day excision of internal hemorrhoidal tumors is practised nearly exclusively by the French surgeons, the operation having been almost entirely abandoned in Great Britain and this country. And considering the seat and nature of the disease, it seems to me exceedingly strange how any intelligent and honest surgeon can conscientiously advise a patient to submit to so hazardous a procedure—except in a few rare cases—

when other and less dangerous means have proved so much more successful. The great danger, as the reader will of course understand, is hemorrhage; but to what extent this accident has proved fatal it is impossible to ascertain, as surgeons generally, and more particularly those of France, where, as just stated, this operation has been most extensively employed, are not in the habit of making public their unsuccessful cases of any kind; a fact that cannot be too much regretted in view of the great benefit that such reports would undoubtedly confer upon the profession. That fatal effects however, from this cause have frequently followed the operation, we have some of the best authority for believing. Thus honest old BOYER in his immortal work entitled "*Traité des Maladies Chirurgicales*," states that instances of this kind have been frequently reported. His son. Baron PHILIP BOYER, admits the loss, from this cause, of one patient out of sixteen upon whom he performed the operation, also one from tetanus, and three others who, to use his own language, "*succombèrent à l'adynamie qu'avaient préparée les pertes abondantes de sang*,"—in all, five cases out of sixteen. J. L. PETIT tells of a young surgeon who imprudently extirpated some hemorrhoidal tumors, which he supposed to be external but which proved to be internal. Frightened by the violent hemorrhage he plugged the rectum with charpie, and no blood being thus allowed to escape externally, he thought he had arrested it; but the patient died and PETIT found the rectum and nearly the whole of the colon full of semi-fluid venous blood. Sir ASTLEY COOPER says, that in the early part of his career he was a strong advocate for excision, but was ultimately induced to abandon it from having met with three fatal cases, two of the three resulting from hemorrhage, and the other from peritoneal inflammation. And it is worthy of remark that in each of the cases that died from hemorrhage, but a single tumor was cut off. Sir BENJAMIN BRODIE, although not as unfortunate as to lose any of his patients, records his entire experience in this operation as follows: "In the first one or two cases I found no inconvenience to arise from my altered practice (referring to his adoption of excision for the ligature); but then a case occurred in which the patient lost a great deal of blood; in another case the hemorrhage was so great that the patient nearly died; and then a third case occurred, in which also the patient lost an enormous quantity of blood—so much, that I now only wonder that he did not actually die." Dupuytren records several cases occurring in his own practice in which the hemorrhage was very alarming, but, Frenchman-like, he was not to be deterred by a little accident of this sort from continuing his favorite practice, only he took the precaution ever afterwards, when he thought there was any danger, of touching the cut surface with the actual cautery.

Looking therefore at the situation and morbid anatomy of internal hemorrhoids and at the frequent, alarming and often fatal effects of extirpation by excision, it is my honest opinion that the operation ought to be almost entirely discarded from surgery. Occasional cases occur, it is true, in which it may be very safely employed; cases, for instance, in which the tumors have become solid from coagulation of tneir contents or adhesive inflammation; but even here I think it preferable to let nature accomplish the work by gradual absorption, promoting this process by the local application of stimulants or the introduction of the rectal bougie.

*Ligature.*—The employment of the ligature for the radical cure of internal piles dates back as far as the time of the great father of medicine, the immortal Hippocrates, and it is at this day, *par excellence*, *the* operation. It is just as effectual as excision, and has the great advantage over the latter in being entirely free from the danger of hemorrhage. The great disadvantage that attaches to it is the acute pain by which it is often attended and followed, while, in respect to other accidents, more particularly peritoneal inflammation, there is probably little or no difference between it and excision.

Before proceeding to describe the mode of performing the operation, it is necessary to state the principal circumstances under which it should not be resorted to. 1st. It should not be employed in patients debilitated and broken down in constitution by old age, unless life be seriously threatened by profuse hemorrhage. 2nd. In persons laboring under organic disease of the lungs, heart, or liver. 3rd. In womem during gestation or immediately after parturition. 4th. In cases where there is no protrusion and no serious hemorrhage, until the simpler means stated under the hrad of palliative treatment have been fairly tried. 5th. In periodic cases of vicarious of the catamenial discharge until this latter function has been re-established. 6th. In cases where the tumors are in a state of inflammatory engorgement; and lastly in no case where the system has not been properly and carefully prepared.

The last mentioned point is one to which I desire to call particular attention. No patient laboring under this disease, no matter how healthy he may be in every other respect, should be subjected either to excision or ligature without more or less preliminary treatment. The kind of treatment and the length of time required will, of course, vary in different cases. If the individual be of a plethoric habit of body, accustomed to a full, luxurious diet, and of an active, energetic temperament; a restricted diet, occasional purgation, and partial confinement to the house for ten days or two weeks, will generally accomplish all that is required. If, however, the patient, as is generally the case, be of a relaxed, phlegmatic temperament, debilitated and broken down either by intemperance, dyspepsia, frequent losses of blood, or any other complaint of long standing, a resort to ferruginous tonics, a liberal, well selected allowance of food, gentle out-door exercise, &c., will become necessary, and should be continued until a tolerably healthy tone of action is established, even if it should require several weeks or months. But should there be daily loss of a considerable quantity of blood, which cannot be restrained by local astringents until the requisite state of system is produced, the operation may have to be performed at all hazards. But as a general thing the practitioner should make it an inviolable rule always to institute a systematic course of preparation before undertaking the radical cure by either of the methods stated.

The preliminary treatment having been continued as long as may be judged necessary, the night before the day proposed for the operation, the patient should be required to take a dose of cathartic medicine, as for instance, the common compound calomel pill, followed early the next morning by half a pint of citrate of magnesia, or a table-spoonfull of epsom salts. After the medicine has produced three or four tolerably copious stools he should take three-quarters of a grain or a grain of morphia, to be repeated in the course of an hour or two if necessary, to put the bowels completely at rest. When this is accomplished he is ready for the operation.

*Operation.*—The surgeon having provided himself with four or five stout silk ligatures, which should be well waxed to prevent slipping, a tenaculum, a pair of scissors, and a competent assistant, requests the patient to make violent expulsive efforts over a vessel of warm water, in order to protrude the parts as far as possible. When this is done he places him upon the floor upon his knees and elbows, inserts the tenaculum through the largest of the tumors as near the sphincter as may be, and makes gentle traction upon the instrument to steady and draw the tumor out as much as possible without lacerating it; he then commits the tenaculum to the charge of the assistant, encircles the tumor with the ligature beyond the convexity of the instrument, and ties a firm unyielding knot. In this last mentioned procedure some care is required, lest the ligature be drawn so tightly as to divide the tissues; the object is not to cut the tumor off but to strangulate it, and for this purpose, although the thread has to be drawn very firmly, the surgeon should be on his guard not to go beyond the mark. The ligature having been tied, one or both ends may be cut off within a short distance of the knot, although I think it preferable that one should be left hanging out at the anus.

The number of tumors to be tied will vary in different cases although as a general rule three or four will suffice. There is no necessity for tying all of them; usually about two-thirds or three-fourths of the whole number are enough; the remainder will become obliterated by the effusion of lymph consequent upon the inflammatory action, which necessarily follows the operation.

After all the ligatures have been tied, and one end of each cut off, the parts should be returned very gently, and the patient put to bed. If much pain is complained of, another grain of morphia will be required, together with the local application of cloths wrung out of cold water, or, what is often more agreeable to the patient's feelings, a bottle of hot water placed between the thighs and pressed up in contact with the anus. As a common rule it is advisable to administer a full opiate immediately after the operation whether there be much pain or not, and to continue its use in sufficient quantities to keep up a state of slight narcotism for three or four days; it insures perfect quietude of the bowels—a point very much to be desired—controls inflammatory action, and is the safest possible guaranty against the occurrence of peritonitis. The bowels should on no account be allowed to act under four or five days at least, to prevent which, in addition to using the morphia or other preparation of opium, the patient should be subjected to a rigorous diet, consisting only of a little mush and milk, chicken broth, crackers, or other bland, unirritating articles. At the end of this time a saline cathartic may be given, aided by large injections of warm water, and after free evacuation the morphia should be again resorted to for three or four days, and again a gentle cathartic. At the end of a week or ten days, if everything is favorable, the opiate may be suspended, the bowels kept gently open by the use of sulphur water, citrate of magnesia, or some other mild laxative, and the patient allowed to sit up from time to time throughout the day.

Most of the ligatures accompanied by more or less clotted blood, the debris of the strangulated tumors, and a considerable quantity of stringy mucus, are usually discharged with the first evacuation of fœcal matter, if this has been delayed for six or eight days. If any should remain longer than this time, gentle traction may be made daily upon the ends hanging from the anus, but not sufficient to endanger laceration or to produce pain; this will promote their detachment and they will generally be discharged a day or two thereafter. Another circumstance which occurs at this first and often at several subsequent evacuations of the bowels and well deserves to be mentioned, is the re protrusion of the parts in apparently as bad a condition as ever. This very naturally alarms the patient, and may lead the inexperienced surgeon to suppose that the operation has been entirely unsuccessful; but it need not, for it results simply from the congested and œdematous condition of the parts resulting from the inflammation, and will gradually disappear in the course of ten days or two weeks. During this period, the patient is also liable to suffer from more or less strangury which should be alleviated as much as possible by cloths wrung out of warm water applied over the hypogastrium, the free use of diluent drinks, or mild unirritating diuretics, aided if necessary, by an occasional injection of starch and laudanum, or a suppository of opium. If violent tenesmus, accompanied by constitutional irritation, should occur in spite of the internal use of morphia, it may become necessary to apply six or eight leeches around the anus, or even to take blood from the arm; but very generally an injection of cold water to wash out the lower bowel, followed by the laudanum and starch above directed, is all that is required.

When the tumors are very large, it has been advised to make use of a large curved needle armed with a double ligature, and carried through the base of the tumor, so that the two threads may be tied on opposite sides, each encircling only one half of the swelling; but this, I imagine, is rarely necessary. Under these circumstances a part only of the tumor need be ligated, following the plan already described, for the unembraced portion is almost sure to be obliterated by the subsequent inflammation.

Almost the only, and certainly the greatest, danger to be apprehended from the application of the ligature to internal hemorrhoids, is peritoneal inflammation. This, however, is comparatively a rare occurrence, especially if the patient's system has been properly prepared for the operation, and opinm or morphia freely employed as heretofore advised. If this accident should happen, it should be treated upon the general principles, which are familiar to all practitioners. The great remedy, however, in my opinion, is opium or some of its preparations, given to the effect of partial narcotism, and kept up for several successive days.

In regard to the other modes, namely, the seton, introduction of hot needles, &c., proposed for the radical cure of internal hemorrhoids, I know nothing, nor do I believe that they have ever been put in practice. I mention them simply because they are applicable to varicose tumors occurring in other parts of the body, and might properly be employed here in very bad cases in which the surgeon wished to make use of milder means before resorting to the ligature.

The *preventive treatment* of internal hemorrhoids is demanded in persons who, from constitution or occupation, are predisposed to the disease, and in all cases after a cure has been effected. It is a well known fact that the disease is very apt to recur in persons who do not take the necessary precaution, and to all who have been so fortunate as to be relieved, this is especially necessary. The means to be made use of are those pointed out under the head of *palliative treatment.* The most important of these is a removal from all predisposing causes, a constant attention to the state of the bowels, and the use of a properly constructed water-closet.

Louisville, July 31, 1854.

